# Inhibition of Cell Growth and Induction of Apoptosis by *Antrodia camphorata* in HER-2/*neu*-Overexpressing Breast Cancer Cells through the Induction of ROS, Depletion of HER-2/*neu*, and Disruption of the PI3K/Akt Signaling Pathway

**DOI:** 10.1155/2012/702857

**Published:** 2012-06-03

**Authors:** Chuan-Chen Lee, Hsin-Ling Yang, Tzong-Der Way, K. J. Senthil Kumar, Ying-Chen Juan, Hsin-Ju Cho, Kai-Yuan Lin, Li-Sung Hsu, Ssu-Ching Chen, You-Cheng Hseu

**Affiliations:** ^1^Department of Health and Nutrition Biotechnology, Asia University, Taichung 41354, Taiwan; ^2^Institute of Nutrition, China Medical University, Taichung 40402, Taiwan; ^3^Department of Life Sciences, China Medical University, Taichung 40402, Taiwan; ^4^Department of Cosmeceutics, College of Pharmacy, China Medical University, Taichung 40402, Taiwan; ^5^Department of Medical Research, Chi-Mei Medical Center, Tainan 71079, Taiwan; ^6^Institute of Biochemistry and Biotechnology, Chung Shan Medical University, Taichung 40402, Taiwan; ^7^Department of Life Sciences, National Central University, Chung Li 32001, Taiwan

## Abstract

Previously, we demonstrated that a submerged fermentation culture of *Antrodia camphorata* (AC) promotes cell-cycle arrest and apoptosis in human estrogen receptor-positive/negative breast cancer cells. However, whether AC is effective against HER-2/*neu*-overexpressing breast cancers has not been thoroughly elucidated. In the present study, we showed that AC exhibited a significant cytotoxic effect against HER-2/*neu*-overexpressing MDA-MB-453 and BT-474 cells. Immunoblot analysis demonstrated that HER-2/*neu* and their tyrosine phosphorylation were inhibited by AC in a dose-dependent manner. An increase in intracellular reactive oxygen species (ROS) was observed in AC-treated cells, whereas antioxidant *N*-acetylcysteine (NAC) significantly prevented AC induced HER-2/*neu* depletion and cell death, which directly indicates that AC-induced HER-2/*neu* depletion and cell death was mediated by ROS generation. Also, AC significantly downregulated the expression of cyclin D1, cyclin E, and CDK4 followed by the suppression of PI3K/Akt, and their downstream effectors GSK-3**β** and **β**-catenin. Notably, AC-treatment induced apoptotic cell death, which was associated with sub-G1 accumulation, DNA fragmentation, mitochondrial dysfunction, cytochrome *c* release, caspase-3/-9 activation, PARP degradation, and Bcl-2/Bax dysregulation. Assays for colony formation also confirmed the growth-inhibitory effects of AC. This is the first report confirming the anticancer activity of this potentially beneficial mushroom against human HER-2/*neu*-overexpressing breast cancers.

## 1. Introduction

Breast cancer is the most common cancer among women in the Western world and is the second leading cause of cancer-related death worldwide [1]. Sporadic breast cancer is the most common solid tumor and kills nearly 40,000 women per year in the United States alone [2]. The current treatment of estrogen-receptor (ER-) positive tumors (>60% of breast cancers) primarily relies on surgery to remove gross tumors followed by treatment with drugs that exploit the hormone dependence of these tumors, including aromatase inhibitors and antiestrogens such as tamoxifen [3]. However, those drugs have a moderate effect against certain types of breast cancer cells, such as HER-2/*neu-*overexpressing breast cancers.

The HER-2/*neu* protooncogene is the second member of the epidermal growth factor receptor (HER, also known as ErbB) family, which consists of four receptors: EGFR (HER-1/ErbB1), HER-2 (ErbB2), HER-3 (ErbB3), and HER-4 (ErbB) [4]. More than 30% of breast cancers were found to have HER-2/*neu* overexpression, which is considered a negative prognostic factor and a predictive marker of resistance to tamoxifen therapy. Aberrant activation of the HER-2 receptor is closely associated with the development and severity of many cancers, including human breast cancers [5, 6]. HER-2/*neu *expression is associated with increased metastatic potential and resistance to chemotherapeutic agents, suggesting that the enhanced tyrosine kinase activity of HER-2/*neu *may play a critical role in the initiation, progression, and outcome of human breast tumors [7, 8]. Activation of receptor tyrosine kinases (185 kDa), which are transmembrane receptors with an intrinsic ability to phosphorylate tyrosine residues in their cytoplasmic domains, such as PI3K and Akt, results in the activation of nuclear transcription factors that induce cell growth and inhibit apoptosis [7]. Therefore, targeting HER-2/*neu* has been the main focus in breast cancer treatment, although the inhibition of HER-2/*neu* has become an increasingly important therapeutic target for human breast cancers.

*Antrodia camphorata* (AC), an indigenous medicinal mushroom that is popularly known as “Niu Cheng Zhi” in Taiwan, is a newly discovered basidiomycete of the family Polyporaceae that only grows in the inner sap of the native Taiwanese tree* Cinnamomum kanehira* Hay (*Lauraceae*) [9]. AC has been used in traditional Chinese medicine for the treatment of food poisoning, drug intoxication, diarrhea, abdominal pain, hypertension, skin irritation, and cancer [10]. A wide range of biological activities have been ascribed to AC, including anticancer, antioxidant, hepatoprotective, antihypertensive, antihyperlipidemic, immunomodulatory, and anti-inflammatory activities [11–13]. AC has low toxicity and is a nonmutagenic beneficial mushroom that efficiently reduces the risk of various cancers. Notably, AC has been shown to inhibit antiproliferation and/or induce apoptosis in hormone-dependent MCF-7 and hormone-independent MDA-MB-231 breast carcinoma cell lines [13–17]. However, MCF-7 and MDA-MB-231 express only basal levels of HER-2/*neu*. In this study, we investigated the effectiveness of the fermented broth of AC harvested from submerged cultures against two human breast cancer cell lines with high levels of HER-2/*neu *expression. We demonstrated the AC-mediated growth inhibition and apoptotic induction of HER-2/*neu*-overexpressing MDA-MB-453 and BT-474 cells through intracellular ROS generation, suppression of the HER-2*/neu *signaling cascade, and disruption of the PI3K/Akt-dependent pathway.

## 2. Materials and Methods

### 2.1. Reagents

 Dulbecco's Modified Eagle's medium (DMEM), nutrient mixture F-12, fetal bovine serum (FBS), glutamine, and penicillin/streptomycin were obtained from GIBCO BRL (Grand Island, NY). 3-(4,5-dimethylthiazol-2-yl)-2,5-diphenyltetrazolium bromide (MTT), *N*-acetylcysteine (NAC), p-iodonitrotetrazolium violet, FITC, and NH_4_Cl were purchased from Sigma-Aldrich Chemical Co. (St. Louis, MO). Antibodies against p-tyrosine, cyclin E, p27^KIP^, p21^CIP^, CDK1, CDK2, cytochrome *c*, Bcl-2, Bax, PARP, and *β*-actin were purchased from Santa Cruz Biotechnology, Inc. (Heidelberg, Germany). Antibodies against HER-2/*neu* (p185), p-PI3K, PI3K, p-Akt, Akt, *β*-catenin, GSK-3*β*, caspase-3, caspase-9, cyclin D1, and CDK4 were obtained from Cell Signaling Technology, Inc. (Danvers, MA). 4′,6-diamidino-2-phenylindole dihydrochloride (DAPI) and the Akt inhibitor LY294002 were purchased from Calbiochem (La Jolla, CA). The proteasome inhibitor MG132 was purchased from Bio Vision, Inc. (Mountain View, CA). All other chemicals were reagent grade or HPLC grade and were supplied by either Merck & Co., Inc. (Darmstadt, Germany) or by Sigma-Aldrich (St. Louis, MO).

### 2.2. Preparation of the Fermented Culture Broth of AC from Submerged Cultures

The AC culture was inoculated onto potato dextrose agar and incubated at 30°C for 15–20 days. The whole colony was subsequently added to a flask containing 50 mL sterile water. After homogenization, the fragmented mycelial suspension was used as an inoculum. The seed culture was prepared in a 20 L fermenter (BioTop) agitated at 150 rpm with an aeration rate of 0.2 vvm at 30°C. A five-day culture of 15 L mycelium inoculum was inoculated into a 250 L agitated fermenter (BioTop). The fermentation conditions were the same as those used for the seed fermentation, but the aeration rate was 0.075 vvm. The fermentation product was harvested at hour 331 and poured through a nonwoven fabric on a 20-mesh sieve to separate the deep-red fermented culture broth and the mycelia; the culture broth was centrifuged thereafter at 3000× g for 10 min followed by passage through a 0.22 *μ*m filter. The culture broth was concentrated under vacuum and freeze-dried to a powder. The yield of dry matter from the culture broth was 18.4 g/L. The experiments were performed with 2~4 different batches of AC fermented culture [18]. To prepare the stock solution, the powder samples were solubilized with DMEM containing 1% FBS (pH 7.4). The stock solution (1.6 mg/mL) was stored at −20°C before its anticancer properties were evaluated. We refer to the fermented culture broth of* A. camphorata* as AC throughout the paper.

### 2.3. Cell Culture

 The human breast cancer cell lines MDA-MB-453 and BT-474, which endogenously overexpress the HER-2/*neu *oncogenes, were used in this study. The cell lines were obtained from the American Type Culture Collection (ATCC, Manassas, VA), and cells were grown in DMEM/F12 supplemented with 10% heat-inactivated FBS, 2 mM glutamine, and 1% penicillin-streptomycin-neomycin at 37°C in a humidified incubator with 5% CO_2_. Cultures were harvested and monitored for changes in cell number by counting cell suspensions using a hemocytometer with a phase contrast microscope.

### 2.4. Cell Viability Assay

 Cell viability was monitored by the colorimetric MTT assay. Briefly, cells (2.5 × 10^5^ cells/well in a 24-well plate) were treated with AC (40–240 *μ*g/mL) for 24 h. Next, 0.5 mg/mL MTT in phosphate-buffered saline (PBS, 400 *μ*L) was added to each well and incubated at 37°C for 4 h. The MTT-generated violet farmazan crystals were dissolved in 10% SDS (400 *μ*L/well), and the absorbance was measured at 570 nm (A_570_). Cell viability (%) was calculated as (A_570_ of treated cells/A_570_ of untreated cells) × 100.

### 2.5. Determination of Apoptosis

Apoptotic cell death was measured using terminal deoxynucleotidyl transferase-mediated dUTP-fluorescein nick end-labeling (TUNEL) with a fragmented DNA detection kit (Roche, Mannheim, Germany). Cells (2 × 10^4^ cells/well) were seeded on DMEM/F-12 medium with 10% FBS in glass (eight-well) Tek chambers (Nunc, Denmark) and treated with various concentrations of AC (40–240 *μ*g/mL) for 24 h. After AC treatment, cells were washed with PBS twice, fixed in 2% paraformaldehyde for 30 min, and permeabilized with 0.1% Triton X-100 for 30 min at room temperature. The cells were then incubated with TUNEL reaction buffer in a 37°C humidified chamber for 1 h in the dark, rinsed twice with PBS, and incubated with DAPI (1 mg/mL) at 37°C for 5 min; stained cells were visualized by fluorescence microscopy.

### 2.6. Western Blot Analysis

MDA-MB-453 or BT-474 cells (1.5 × 10^6^ cells/10 cm dish) were incubated with various concentrations of AC for 24 h. After incubation, the cells were washed once in PBS, detached, pooled, and centrifuged at 1500× g for 5 min. The cell pellets were subsequently suspended in 100 *μ*L lysis buffer (10 mM Tris-HCl, pH 8.0, 320 mM sucrose, 1% Triton X-100, 5 mM EDTA, 2 mM dithiothreitol, and 1 mM phenylmethylsulfonyl fluoride). The suspensions were kept on ice for 20 min and centrifuged at 15000× g for 30 min at 4°C. Total protein content was determined with the Bio-Rad protein assay reagent (Bio-Rad, Hercules, CA) using BSA as a standard. Protein extracts were reconstituted in sample buffer (62 mM Tris-HCl, 2% SDS, 10% glycerol, 5%  *β*-mercaptoethanol), and the mixture was boiled at 97°C for 5 min. Equal amounts (50 *μ*g) of denatured protein samples were loaded into each lane, separated by SDS-PAGE on an 8–15% polyacrylamide gradient gel and transferred onto polyvinylidene difluoride membranes overnight. The membranes were blocked with 5% nonfat dried milk in PBS containing 1% Tween-20 for 1 h at room temperature and subsequently incubated with primary antibodies for 2 h and either horseradish peroxidase-conjugated goat anti-rabbit or anti-mouse antibodies overnight. Blots were visualized on ImageQuant LAS 4000 mini (Fujifilm) system with SuperSignal West Pico chemiluminescence substrate (Thermo Scientific, IL).

### 2.7. Fluorescence Imaging of HER-2/*neu*

MDA-MB-453 and BT-474 cells (2 × 10^4^ cells/well) were cultured in DMEM/F-12 medium with 10% FBS in glass eight-well Tek chambers. After AC treatment, the cells were fixed in 2% paraformaldehyde for 15 min, permeabilized with 0.1% Triton X-100 for 10 min, washed and blocked with 10% FBS in PBS, and incubated for 2 h with an anti-HER-2*/neu* primary antibody in 1.5% FBS. The cells were subsequently incubated with a FITC-conjugated secondary antibody for 1 h in 6% bovine serum albumin followed by staining with 1 *μ*g/mL DAPI for 5 min. The stained cells were washed with PBS and visualized using a fluorescence microscope at 400x magnification.

### 2.8. Colony Formation Assay

 Anchorage-independent growth was determined by colony formation in soft agar [19]. The assay was performed in 6-well plates (1 × 10^4^ cells/well) with a base layer containing 0.5% agar in DMEM containing 10% FBS, 1 mM glutamine, 100 units penicillin, and 100 *μ*g/mL streptomycin. This layer was overlaid with a second layer of 1 mL 0.35% agar (in DMEM containing 10% FBS, 1 mM glutamine, 100 units of penicillin, and 100 *μ*g of streptomycin) with a suspension of 1 × 10^4^ cells/well. Fresh medium with AC (40–240 *μ*g/mL) was then added to the plates every 72 h. The plates were incubated at 37°C for 3 weeks, and the tumor colonies were analyzed with a microscope. Colonies with a diameter greater than 0.2 mm were counted.

### 2.9. Measurement of ROS Generation

 Intracellular ROS accumulation was detected by fluorescence microscopy with 2′,7′-dihydrofluorescein-diacetate (DCFH-DA). Cells (1 × 10^5^ cells/12 wells) were cultured in DMEM/F-12 supplemented with 10% FBS. To evaluate the generation of ROS in a time-dependent manner, cells were treated with 160 *μ*g/mL AC for 0, 1, 5, 10, and 30 min. After treatment for the indicated time points, the cells were then incubated with 10 *μ*M DCFH-DA in culture medium at 37°C for 30 min. The acetate groups on DCFH-DA were removed by an intracellular esterase, trapping the probe inside the MDA-MB-453 cells. After loading, the cells were washed with warm PBS buffer. The production of ROS can be measured by changes in fluorescence due to the intracellular accumulation of dichlorofluorescein (DCF) caused by oxidation of DCFH. Intracellular ROS, as indicated by DCF fluorescence, was measured by fluorescence microscopy (Olympus 1 × 71 at 200x magnification).

### 2.10. Statistical Analysis

 The results are presented as the mean ± standard deviation (mean ± SD). All study data were analyzed using analysis of variance followed by Dunnett's test for pairwise comparison. An asterisk indicates that the experimental values are significantly different from those of the control (*P < 0.05).

## 3. Results

### 3.1. AC Treatment Inhibits Proliferation of HER-2/*neu*-Overexpressing Breast Cancer Cells

**Figure 1:**
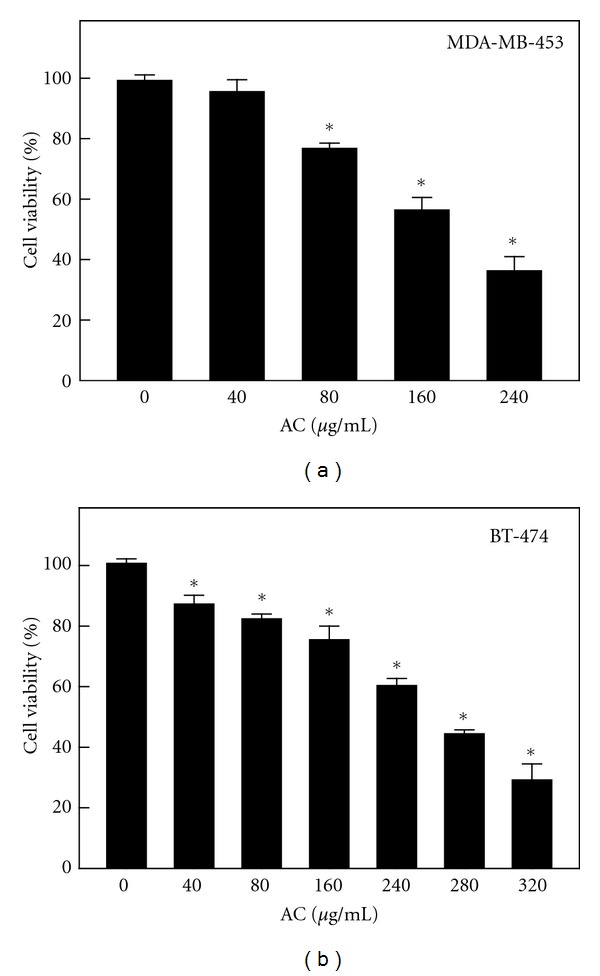
Inhibitory effect of AC on the proliferation of the HER-2/*neu*-overexpressing breast cancer cell lines MDA-MB-453 and BT-474. After incubation with different concentrations of AC (40–320 *μ*g/mL) at 37°C for 24 h, the effect on (a) MDA-MB-231 and (b) BT-474 cell growth was examined by MTT assay. The number of viable cells after treatment is expressed as a percentage of the vehicle-only control, which was arbitrarily assigned 100%. The results are presented as the mean ± SD of three independent assays. *Significant difference in comparison to the control group (*P* < 0.05).

 To evaluate the biological activity of AC in terms of cell proliferation, cells were treated with various concentrations of AC for 24 h. To varying extents, a dose-dependent increase in the rate of growth inhibition was observed with 40–320 *μ*g/mL of AC (Figure 1). AC treatment for 24 h resulted in a significant (*P* < 0.05) cytotoxic effect on both HER-2/*neu*-overexpressing MDA-MB-453 and BT-474 breast cancer cells with an IC_50_ of 220 and 240 *μ*g/mL, respectively (Figures 1(a) and 1(b)). At 240 *μ*g/mL for 24 h, AC inhibited >60% of growth in MDA-MB-453 and >40% in BT-474 HER-2/*neu*-overexpressing breast cancer cells (Figures 1(a) and 1(b)). Therefore, treatment of HER-2/*neu*-overexpressing breast cancer cells with AC decreases their rate of proliferation.

### 3.2. AC Treatment Modulates HER-2/*neu* Protein Expression through the Inhibition of Tyrosine Phosphorylation

**Figure 2:**
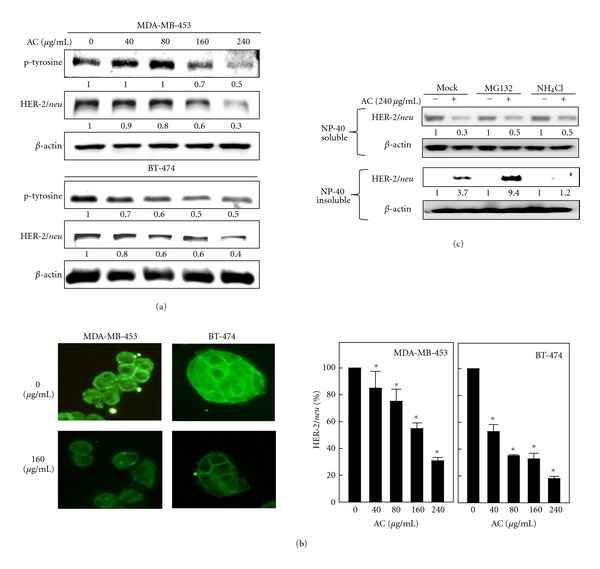
Inhibitory effect of AC on tyrosine phosphorylation and HER-2/*neu* depletion in HER-2/*neu*-overexpressing human breast cancer cell lines. (a) MDA-MB-453 or BT-474 cells were incubated with or without AC (40–240 *μ*g/mL) at 37°C for 6–24 h. Immunoblotting was performed to measure levels of the HER-2/*neu* protein and tyrosine phosphorylation. The proteins (50 *μ*g) in each sample were resolved by 8–15% SDS-PAGE, with *β*-actin serving as a control. (b) Changes in the subcellular distribution of HER-2/*neu *after a 24-h exposure to AC. MDA-MB-453 and BT-474 cells were grown on coverslips and treated with or without AC (40–240 *μ*g/mL). Cells were fixed with 4% paraformaldehyde and stained with a HER-2/*neu *antibody followed by a fluorescein isothiocyanate-conjugated secondary antibody (green). The subcellular distribution was photographed by fluorescence microscopy. (c) MDA-MB-453 cells were pretreated with MG132 (5 *μ*M) or NH_4_Cl (10 mM) for 30 min followed by AC (240 *μ*g/mL) for 8 h, and the NP-40-soluble and NP-40-insoluble cell lysates were prepared and assessed by immunoblotting with antibodies against HER-2/*neu* and *β*-actin. Relative changes in protein bands were measured using densitometric analysis; the control was 1.0-fold, as shown immediately below the gel data. The results are presented as the mean ± SD of three independent experiments. *Significant difference in comparison to the control group (*P* < 0.05).

 Activation of the HER-2/*neu* network leads to autophosphorylation of the C-terminal tyrosine and the recruitment to these sites of cytoplasmic signal transducers that regulate cellular processes, such as proliferation, inhibition of apoptosis, and transformation. Therefore, we sought to examine whether treatment with AC could reduce this basal tyrosine kinase phosphorylation. MDA-MB-453 and BT-474 human breast cancer cells were treated with 40, 80, 160, and 240 *μ*g/mL of AC for 24 h. The total cell lysates were isolated and subjected to Western Blot analysis using HER-2/*neu* and phosphotyrosine-specific HER-2/*neu* antibodies. As shown in Figure 2(a), treatment of MDA-MB-453 and BT-474 cells with 40–240 *μ*g/mL AC for 24 h resulted in a substantial decrease in HER-2/*neu* tyrosine phosphorylation. AC treatment similarly reduced basal HER-2/*neu* levels in both cell lines (Figure 2(a)). Taken together, these findings indicate that AC reduces the basal tyrosine kinase phosphorylation and constitutive activation of HER-2/*neu* receptors in HER-2/*neu*-overexpressing breast cancer cells.

To confirm the Western Blot data summarized in Figure 2(a), immunofluorescence images of HER-2/*neu* expression were examined. Representative images of untreated MDA-MB-453 and BT-474 cells compared with cells treated with AC are shown in Figure 2(b). AC-treated cells exhibited lower levels of immunofluorescence at the plasma membrane, and fluorescence was replaced by diffuse cytoplasmic punctate staining. At 160 *μ*g/mL, AC caused a significant inhibition (*P* < 0.05) and localization of membrane-bound HER-2/*neu* in MDA-MB-453 and BT-474 cells (Figure 2(b)).

To delineate better the mechanism of AC-mediated HER-2/*neu* downregulation, we examined the effect of AC on HER-2/*neu* mRNA levels. When comparing protein and mRNA levels, HER-2/*neu* protein levels decreased in a dose-dependent manner after AC treatment, whereas HER-2/*neu* mRNA levels in MDA-MB-453 and BT-474 cells were not significantly decreased by AC treatment, even after 24 h (data not shown). Moreover, addition of cycloheximide, a translation inhibitor, does not alter the effect of AC on the immunofluorescence pattern of HER-2/*neu* protein levels (data not shown), indicating that AC treatment did not affect HER-2/*neu *mRNA levels or change the rate of *de novo *synthesis of HER-2/*neu*. Taken together, the present data suggest that the AC-associated reduction of HER-2/*neu* expression may not involve a posttranscriptional mechanism.

### 3.3. AC Treatment Promotes HER-2/*neu* Proteasomal Degradation in MDA-MB-453 Cells

 To examine the role of proteolysis in AC-mediated HER-2/*neu* downregulation, we used the proteasome inhibitor MG132 or the lysosome inhibitor NH_4_Cl. In the absence of MG132 or NH_4_Cl, AC treatment significantly reduced HER-2/*neu* levels in the detergent (NP-40)-soluble fractions (Figure 2(c)). Cotreatment with the proteasome inhibitor MG-132 resulted in accumulation of insoluble (aggregated) forms of Her-2*/neu* protein in cell lysates (Figure 2(c)). Unlike MG-132, the lysosomal inhibitor NH_4_Cl did not prevent the downregulation of Her-2*/neu* protein during treatment with AC. These data suggest that proteasomal activity was critically involved in AC-induced HER-2/*neu* degradation in human breast cancer MDA-MB-453 cells.

### 3.4. AC-Induced Cell Death Was Mediated by Intracellular ROS Generation

**Figure 3:**
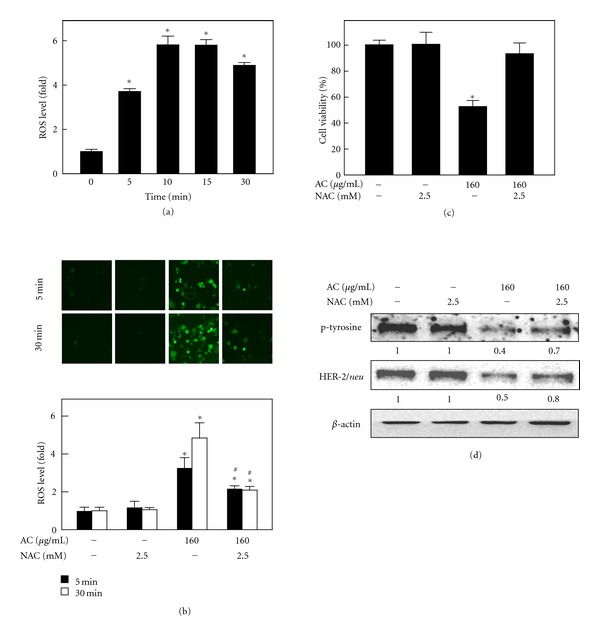
AC-induced ROS generation and its involvement in cell death. (a) MDA-MB-453 cells were treated with AC (160 *μ*g/mL) for 0, 1, 5, 10, 30, and 60 min. The nonfluorescent cell-membrane-permeable probe DCFH-DA was added to the culture medium at a final concentration of 10 *μ*M for 30 min before the end of each experiment. DCFH-DA penetrated the cells, reacted with cellular ROS, and was metabolized into fluorescent DCF, as indicated by DCF fluorescence, which was measured by fluorescence microscopy (200x magnification). The intracellular ROS level was expressed graphically as a relative fold increase of the control. (b)–(d) MDA-MB-453 cells were pretreated with 2.5 mM NAC, an antioxidant, for 1 h followed by with or without AC (160 *μ*g/mL) treatment and quantified intracellular ROS generation (b), cell viability (c), and HER-2/*neu* and p-tyrosine protein levels (d). The photomicrographs shown in this figure are from one representative experiment that was performed in triplicate with similar results. Each value is expressed as the mean ± SD (*n* = 3). ^∗,#^Significant difference between the control and AC-treated group (*P* < 0.05).

We have previously reported that AC treatment caused ROS generation in human breast cancer MCF-7 cells, which is proposed to be one of the early events in the activation of apoptotic signaling. In this study, we also examined the involvement of AC in ROS generation in MDA-MB-453 cells. Fluorescence microscopy with DCFH-DA as a fluorescent probe was performed to estimate the intracellular ROS accumulation in MDA-MB-453 cells. Incubation of cells with AC (160 *μ*g/mL for 0, 1, 5, 10, 30, and 60 min) caused a significant increase in fluorescence, and the maximum level of ROS accumulation (*P* < 0.05) was observed at 5 min after AC treatment (Figure 3(a)). To investigate further whether AC-induced cell death could be linked to ROS generation, *N*-acetylcysteine (NAC), a scavenger of ROS, was used in MDA-MB-453 cells. Cells were simultaneously treated with AC (160 *μ*g/mL) and NAC (2.5 mM) for 1 h. As shown in Figure 3(b), exposure of MDA-MB-453 cells to AC (160 *μ*g/mL) led to 3.7-fold and 5.2-fold increases in the DCF signal compared with control cells at 5 and 30 min, respectively. NAC pretreatment significantly (*P* < 0.05) inhibited the increase in DCF fluorescence by 2-fold at the same time points (Figure 3(b)), which was concomitant with the inhibition of AC-induced cell death in MDA-MB-453 cells (*P* < 0.05) (Figure 3(c)).

The results described above suggested that AC-induced cell death was mediated by intracellular ROS generation in MDA-MB-453 cells (Figure 3(c)). To confirm further the effects of NAC on AC-induced ROS generation and cell death in HER-2*/neu*-overexpressing breast cancer cells, the protein levels of HER-2/*neu* were investigated. As shown in Figure 3(d), AC (160 *μ*g/mL for 24 h) caused a significant decrease in HER-2/*neu *and tyrosine phosphorylation levels by 0.5-fold and 0.4-fold, respectively. However, pretreatment of MDA-MB-453 cells with NAC for 1 h resulted in a significant protection against AC-induced HER-2/*neu* depletion, as well as tyrosine phosphorylation (Figure 3(d)). Taken together, NAC pretreatment downregulates AC-induced ROS generation, cell viability, and HER-2/*neu *expression, which was direct evidence that AC-induced cell death was mediated by ROS generation and/or HER-2/*neu *inhibition.

### 3.5. AC Treatment Inhibited the Activation of PI3K/Akt in HER-2/*neu*-Overexpressing Breast Cancer Cells

**Figure 4:**
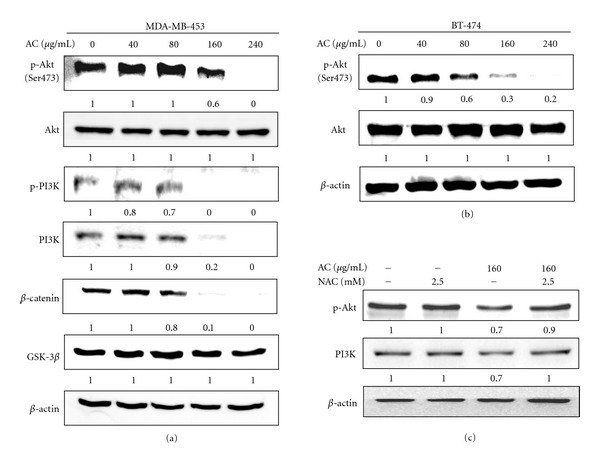
AC treatment suppressed the phosphorylation of PI3K/Akt and GSK-3*β*/*β*-catenin in HER-2/*neu*-overexpressing breast cancer cell lines. (a) MDA-MB-453, (b) BT-474, and (c) 2.5 mM NAC pretreated MDA-MB-453 cells were treated with or without AC (40–240 *μ*g/mL) for 24 h. The levels of phosphorylated PI3K (p-PI3K) and Akt (p-Akt, pSer 473 Akt) were evaluated using phosphorylated antibodies specific to PI3K and Akt in an immunoblot analysis. The total PI3K and Akt levels were assessed as the loading control. The levels of indicated proteins in the cell lysates were analyzed with specific antibodies, and the amount of *β*-actin was used as an internal control for sample loading. The photomicrographs shown in this figure are from one representative experiment that was performed in triplicate with similar results. Relative changes in protein bands were measured using densitometric analysis; the control was 1.0-fold, as shown immediately below the gel data. The results are presented as the mean ± SD of three assays. *Significant difference in comparison to the control group (*P* < 0.05).

 A key mechanism by which HER-2/*neu-*overexpression stimulates tumor cell growth and renders cells chemoresistant involves the HER-2/*neu *receptor. This mechanism involves the PI3K/Akt signaling pathway, and human breast cancer cells with overexpression and amplification of HER-2/*neu *have been shown to make increased use of the PI3K/Akt signaling pathway [7]. We next sought to determine the involvement of HER-2/*neu* in the activation of the PI3K/Akt signaling pathway in MDA-MB-453 and BT-474 cell lines. AC treatment significantly inhibited the phosphorylation of Akt in MDA-MB-453 HER-2/*neu*-overexpressing breast cancer cells in a dose-dependent manner (Figures 4(a) and 4(b)). In addition, we observed that AC treatment significantly inhibited the expression of the Akt upstream kinase, PI3K, in MDA-MB-453 cells (Figure 4(a)). AC caused a similar dose-dependent reduction in Akt phosphorylation in BT-474 cells, whereas the levels of total Akt were unaffected by AC under the same treatment conditions (Figure 4(b)). These data established that AC-induced HER-2/*neu* depletion and growth inhibition may be mediated by the inactivation of PI3K/Akt activity in HER-2/*neu*-overexpressing breast cancer cells. In addition, pretreatment with NAC significantly augmented PI3K/Akt expression, which was suppressed by AC in MDA-MB-453 cells (Figure 4(c)).

### 3.6. AC Treatment Downregulated GSK-3*β* and *β*-Catenin Expression in HER-2/*neu*-Overexpressing Breast Cancer Cells

When PI3K/Akt is active, a number of substrates are activated that involve apoptosis, cell-cycle regulation, and protein synthesis [7]. PI3K/Akt could potentially regulate cell-cycle progression by phosphorylating and inactivating GSK-3*β*, thereby stabilizing nuclear translocation of *β*-catenin and increasing cyclin D1 and Cdk4 transcription [20]. In AC-treated MDA-MD-453 cells, phosphorylated GSK-3*β* levels decreased substantially, while total GSK-3*β* levels increased (Figure 4(a)). This observation suggests that the treatment of cells with AC augmented the activity of GSK-3*β*. Levels of *β*-catenin, a key component of the Wnt signaling pathway that is rapidly degraded *via* polyubiquitination upon phosphorylation by GSK-3*β*, decreased substantially after AC treatment (Figure 4(a)). In conclusion, our data demonstrated that AC may inhibit cell proliferation and the induction of cell death by suppressing GSK-3*β* and the *β*-catenin pathway in HER-2/*neu*-overexpressing breast cancer cells.

### 3.7. AC Treatment Regulates Cell-Cycle Regulatory Proteins in HER-2/*neu*-Overexpressing Breast Cancer Cells

**Figure 5:**
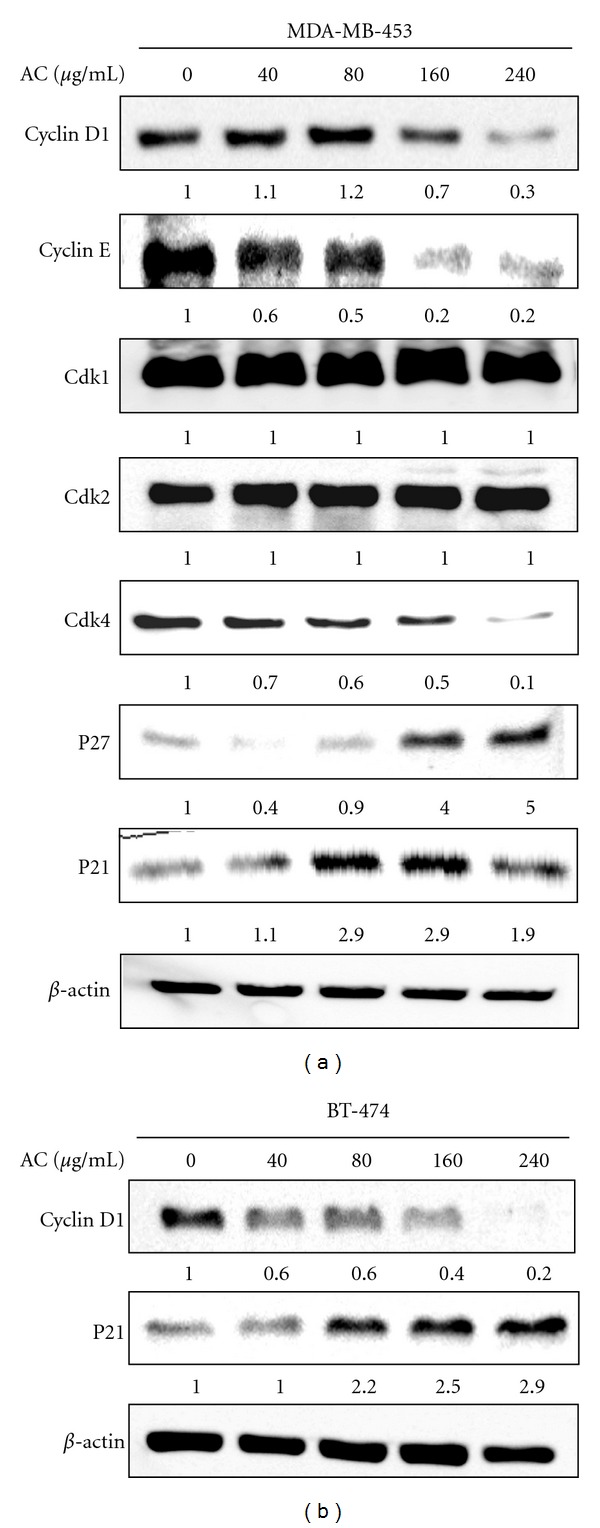
AC altered cell-cycle regulatory proteins in HER-2*/neu*-overexpressing breast cancer cells. (a) MDA-MB-453 and (b) BT-474 cells were treated with or without AC (40–240 *μ*g/mL) for 24 h. Cyclin D1, cyclin E, p21^CIP^, p27^KIP^, Cdk1, Cdk2, Cdk4, and *β*-actin protein levels in MDA-MB-453 cells and cyclin D1, p21^CIP^, and *β*-actin protein levels in BT-474 cells were analyzed by immunoblotting. The proteins (50 *μ*g) in each sample were resolved by 8–15% SDS-PAGE. Relative changes in protein bands were measured by densitometric analysis in which the control was 1.0-fold, as shown immediately below the gel data. The photomicrographs shown in this figure are from one representative experiment that was performed in triplicate with similar results.

 To examine the molecular mechanism(s) and underlying changes in cell-cycle patterns caused by AC treatment, we investigated the effects of various cyclins and Cdks involved in cell-cycle regulation in MDA-MB-453 cells. AC treatment (40–240 *μ*g/mL) for 24 h caused a dose-dependent reduction of cyclin D1 and cyclin E expression in HER-2/*neu*-overexpressing MDA-MB-453 cells (Figure 5(a)). Cyclin D1 serves as the regulatory subunit of Cdk4 and contributes to its stability. Therefore, we assessed the effects of AC on Cdk expression; treatment of MDA-MB-453 cells with AC resulted in a dose-dependent decrease in Cdk4 expression (Figure 5(a)). Nevertheless, there was no change in the Cdk1 and Cdk2 protein levels (Figure 5(a)). These results imply that AC inhibits cell-cycle progression by reducing the levels of cyclin D1, cyclin E, and Cdk4 in MDA-MB-453 cells. In addition, Akt may contribute to the induction of cell-cycle progression by regulating the Cdk inhibitors p27^KIP^ and p21^CIP^ [21]. Previous studies have shown that the modulation of both p27^KIP^ and p21^CIP^ is required for oncogenic growth driven by HER-2 [22]. Both p27^KIP^ and p21^CIP^ protein levels increased dose-dependently in response to AC treatment (Figure 5(a)). A similar pattern of results were also observed in BT-474 cells; AC downregulates cyclin D1 and upregulates p21^CIP^ expression in a dose-dependent fashion (Figure 5(b)).

### 3.8. AC Treatment Promoted Apoptotic Cell Death in HER-2/*neu*-Overexpressing Breast Cancer Cells

**Figure 6:**
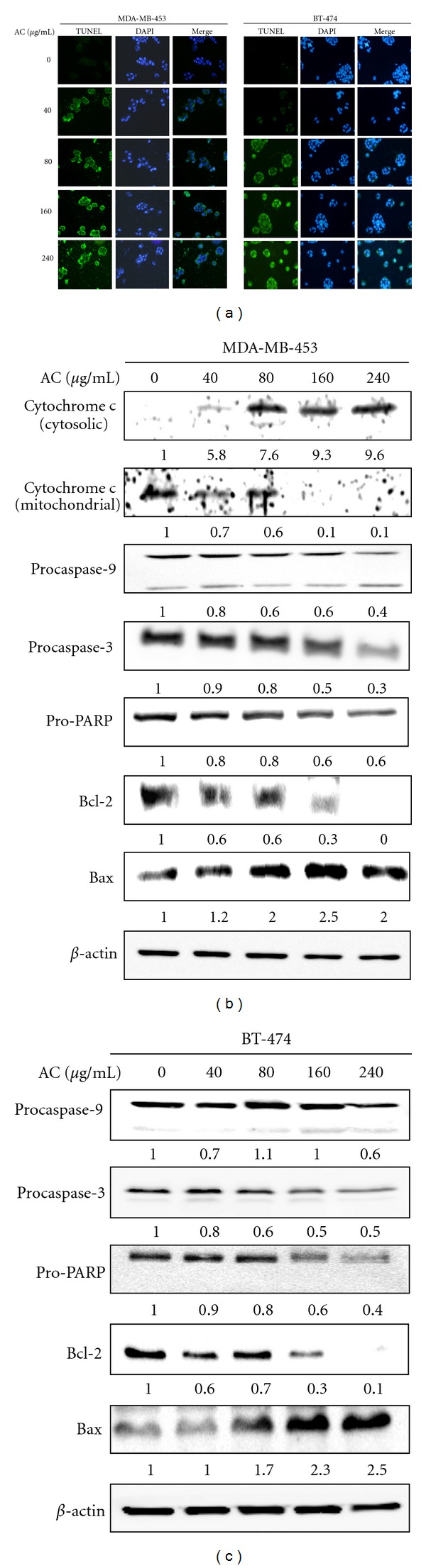
AC induced apoptosis in HER-2/*neu*-overexpressing breast cancer cells. (a) TUNEL assay of MDA-MB-453 and BT-474 cells exposed to AC (40–240 *μ*g/mL for 24 h). The average number of apoptosis-positive cells in microscopic fields (magnification ×400) from three separate samples. (b) Western Blot analysis of apoptosis-related proteins in breast cancer cells exposed to AC (40–240 *μ*g/mL for 24 h). The effects of AC on the protein levels of procaspase-3 and -9, PARP, Bcl-2, Bax, and mitochondrial and cytosolic cytochrome *c* in MDA-MB-453 cells; (c) procaspase-3 and -9, PARP, Bcl-2, and Bax in BT-474 cells. The proteins (50 *μ*g) in each sample were resolved by 8–15% SDS-PAGE with *β*-actin as a control. Relative changes in protein bands were measured by densitometric analysis in which the control was 1.0-fold, as shown immediately below the gel data. The photomicrographs shown here are from one representative experiment repeated two times with similar results.

 A cell survival pathway involving PI3K/Akt is known to play an important role in inhibiting apoptosis in HER-2/*neu*-overexpressing breast cancer cells, which prompted us to examine whether this pathway may play a role in AC-induced apoptosis. Initially, to assess whether AC-induced cell death occurred through apoptotic induction, the DNA fragmentation of an apoptotic biomarker was examined by TUNEL assay. The fragmented DNA was detected by 3′-OH end-labeling of fragmented DNA with dUTP-fluorescein, and TUNEL-positive cells were counted as apoptotic cells.  Figure 6(a) shows the micrographs of characteristic populations of AC (40–240 *μ*g/mL for 24 h)-treated human breast cancer cells. AC treatment initiated DNA fragmentation at 40 and 80 *μ*g/mL in MDA-MB-453 and BT-474 cells, respectively.

We further hypothesized that AC-induced apoptosis may involve mitochondrial pathways. Therefore, mitochondria-mediated apoptosis was evaluated by directly measuring the release of mitochondrial cytochrome *c* into the cytosol by Western Blot analysis. AC significantly induced the aberrant release of mitochondrial cytochrome *c* into cytoplasm after 24 h of treatment (Figure 6(b)), while a decreased amount of mitochondrial cytochrome *c* was observed in the mitochondrial fraction, which was clear evidence that AC caused mitochondrial membrane damage. Cytochrome *c* is reportedly involved in the activation of caspases that trigger apoptosis [15]. Therefore, we investigated the role of caspase-9 and -3 in the cellular response to AC. Immunoblotting showed that treatment of MDA-MB-453 cells with AC significantly induced the proteolytic cleavage of procaspase-9 and -3 into their active forms (Figure 6(b)). PARP-specific proteolytic cleavage by caspase-3 is considered a biochemical characteristic of MDA-MB-453 cells (Figure 6(b)). Incubation of MDA-MB-453 cells with AC caused a dramatic reduction in the level of the antiapoptotic protein Bcl-2 and increased the level of the proapoptotic Bax protein, which heterodimerizes with Bcl-2 to inhibit Bcl-2 activity (Figure 6(b)). These results strongly indicate that AC treatment induced apoptosis through the dysregulation of Bax/Bcl-2. Notably similar results were obtained in the BT-474 human breast cancer cell line in which AC eventually induces apoptosis as evidenced by DNA fragmentation (Figure 6(a)). Furthermore, the AC-induced apoptosis in BT-474 cells was tightly associated with the activation of caspase-9 and -3, and PARP cleavage (Figure 6(c)). AC treatment also resulted in the dysregulation of the Bcl-2/Bax ratio in BT-474 cells (Figure 6(c)). Therefore, we believe that the induction of apoptosis could be a major mechanism of AC-induced growth inhibition in HER-2/*neu*-overexpressing breast cancer cells.

### 3.9. AC Treatment Inhibited Anchorage-Independent Growth of HER-2/*neu*-Overexpressing Breast Cancer Cells

**Figure 7:**
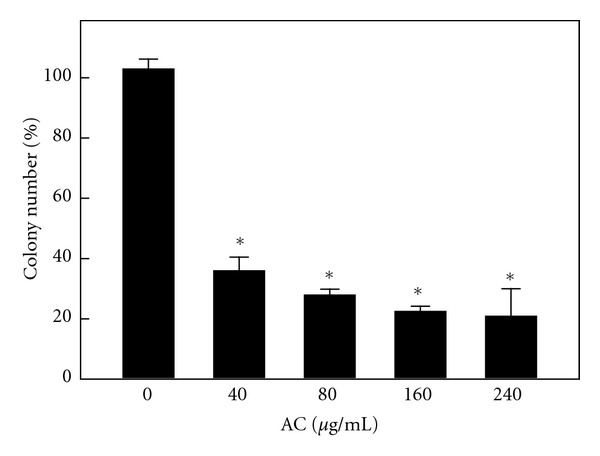
AC inhibits anchorage-independent growth of breast cancer cells. MDA-MB-453 cells were assayed for their ability to proliferate and form colonies in soft agar. Cells were seeded onto 6 cm dishes in culture medium containing 0.35% low-melting agarose over a 0.7% agarose layer in the presence of AC (40–240 *μ*g/mL) or vehicle control (PBS) and incubated for 3 weeks at 37°C. The numbers of colonies >200 *μ*m in size were counted (at a 40x magnification). Colonies were subsequently stained with p-iodonitrotetrazolium violet (1 mg/mL), and colonies larger than 200 *μ*m were counted. The percentage colony formation was calculated by defining the number of colonies in the absence of AC as 100%. The results are presented as the mean ± SD of three independent assays. *Significant difference in comparison to the control group (*P* < 0.05).

 Previous studies demonstrated that human breast cancer cells in which HER-2*/neu* is overexpressed and activated have an increased requirement for a PI3K/Akt-mediated signaling pathway for anchorage-independent growth [7]. We determined whether AC affected anchorage-independent colony growth in soft agar, a property of transformed and tumor cells that is closely correlated with tumorigenesis *in vivo*. Colony formation of MDA-MB-453 cells, which are known to overexpress HER-2/*neu*, was significantly (*P* < 0.05) suppressed (60–70%) by AC relative to the control (Figure 7). Reductions in colony number were accompanied by a reduction in colony size in MDA-MB-453 cells. Therefore, the data indicate that AC treatment suppressed the transformation ability of HER-2*/neu*-overexpressing breast cancer cells.

## 4. Discussion

Our previous studies have shown that *Antrodia camphorata *(AC), an indigenous medicinal mushroom, promoted cell-cycle arrest and apoptosis in human estrogen-responsive MCF-7 and estrogen-nonresponsive MDA-MB-231 breast cancer cells *in vitro* and *in vivo* and that both of these cell lines express basal levels of HER-2/*neu* [13–16, 23]. These effects were only observed in breast cancer cells and not in healthy HBL100 breast cells [24]. This finding indicated that the AC was differentially cytotoxic toward different breast cancer cell lines without exerting harmful effects on normal cells at higher concentrations.

In this study, AC-mediated inhibition of cell proliferation and induction of apoptosis was observed in HER-2/*neu*-overexpressing human breast cancer cells. We showed that AC treatment efficiently inhibited the growth of MDA-MB-453 and BT-474 cells with an IC_50_ value of 220 and 240 *μ*g/mL, respectively. We also demonstrated that exposure of the HER-2*/neu*-overexpressing breast cancer cells to AC resulted in the induction of apoptotic cell death mediated by ROS generation, HER-2*/neu* depletion, and downregulation of PI3K/Akt signaling cascades. These data indicate that the beneficial mushroom may be used as a possible chemopreventive or chemotherapeutic agent against human breast cancers.

Overexpression of human epidermal growth factor receptor-2 (HER-2/*neu*), a 185-kDa transmembrane kinase, was frequently observed in breast cancer cells and has a poor clinical diagnosis. Indeed, agents that reduce HER-2/*neu* activity may be a potential target for breast cancer treatment. Among positive regulators of proliferation, HER-2/*neu* was found to be a complement protooncogene that regulates tumor progression in a variety of human cancers, including breast cancer. Depletion of HER-2/*neu *in HER-2/*neu*-overexpressing human breast cancer cells arrested cell proliferation and activated apoptosis [7]. Trastuzumab (Herceptin), a humanized antibody that targets the extracellular domain of HER-2/*neu*, has become a commercialized drug for the treatment of HER-2/*neu*-overexpressing early-stage and metastatic breast cancers. However, when used as a single agent, trastuzumab is beneficial only in 15–30% of HER-2/*neu* breast cancer patients, which can be significantly increased to 50–80% by the addition of chemotherapeutic drugs [25]. The major observation reported in this study is that AC treatment effectively downregulates HER-2/*neu* protein expression in HER-2/*neu*-overexpressing MDA-MB-453 and BT-474 human breast cancer cells. It has been previously reported that quercetin, a tyrosine kinase inhibitor, eventually blocked HER-2/*neu* expression by inhibiting the phosphorylation of tyrosine kinase in HER-2/*neu*-overexpressing SK-Br3 breast cancer cells [8]. AC, which has also been reported to be a transmembrane tyrosine kinase inhibitor [26], inhibits the tyrosine kinase activity of HER-2/*neu* and induces HER-2/*neu* degradation by the proteasome when inhibition of protein degradation by MG-132 leads to the accumulation of the NP-40-insoluble form of HER-2/*neu*. AC-induced growth inhibition increases the susceptibility of HER-2/*neu*-overexpressing cancer cells. These data indicated that AC may be a promising anticancer agent for human breast cancers.

Many anticancer drugs have been suggested to generate ROS by causing oxidative stress and induce apoptosis in cancer cells, while many inhibitors of apoptosis have antioxidant activity [27]. Indeed, factors that cause or promote oxidative stress, such as ROS production, lipid peroxidation and the downregulation of antioxidant genes, which is characterized by reduced glutathione levels and the reduced transcription of superoxide dismutase, catalase, and thioredoxin, have been shown to be involved in apoptotic processes [27]. Moreover, ROS have also been reported to regulate the activity of certain enzymes involved in the cell-death pathway by inducing mitochondrial dysfunction [28]. The results are consistent with the finding of this study that AC induced growth inhibition and ROS generation in HER-2*/neu*-overexpressing breast cancer cells, indicating that ROS production was probably the major cause of cell death. Also, ROS serve as modulators of proteins, lipid kinases, phosphates, membrane receptors, and transcription factors [29]. Also, ROS generation activates tyrosine kinase by generating growth factors through the cleavage of matrix metalloproteinase [29]. By contrast, AC-induced ROS generation significantly inhibited HER-2/*neu* receptor tyrosine phosphorylation, as evidenced by the inhibition of endogenous HER-2/*neu* receptor tyrosine kinase phosphorylation by AC treatment. Pharmacologically or genetically blocking ROS generation with the antioxidant NAC significantly prevented AC-induced HER-2/*neu *degradation and tyrosine phosphorylation, which was followed by cell growth inhibition.

Previous studies demonstrated that the dysregulation of the PI3K/Akt signaling pathway leads to cancer progression [30]. The PI3K/Akt signaling pathway and its downstream transcription factors have been studied in detail to determine their role in cell proliferation, survival, cell-cycle control, and other cellular functions [31]. In numerous cell types, PI3K/Akt induces survival and suppresses apoptosis induced by a variety of stimuli, including growth-factor withdrawal and loss of cell adhesion [32]. Zheng et al. reported that the overexpression of the HER-2/*neu* gene can activate the PI3K/Akt pathway without exogenous ligand stimulation, and PI3K/Akt pathway activation was also reported to delay apoptosis [33]. We found that treatment with AC had an inhibitory effect on the steady-state levels of total PI3K protein, and its downstream effector, Akt phosphorylation, was inhibited, indicating that the disruption of Akt signaling/Akt inactivation plays a functional role in AC-mediated apoptosis in HER-2/*neu*-overexpressing breast cancer cells. Our present data also suggested that AC-mediated inhibition of cyclin D1/E is directly proportional to the suppression of HER-2/*neu* and PI3K/Akt in human breast cancer cells. Taken together, these results suggest that HER-2*/neu* may regulate cellular cyclin D1/E *via* the PI3K/Akt pathway, implying that PI3K/Akt signaling predominantly contributes to cell-cycle progression.

In the present study, we also demonstrated that AC treatment remarkably downregulates *β*-catenin expression through the upregulation of its negative regulator, GSK-3*β*. The AC-induced increase in GSK-3*β* may contribute to its effects on Wnt/*β*-catenin pathway inhibition. Akt kinase has been shown to phosphorylate several key substrates that regulate protein translation [34] and the phosphorylation of its substrate, GSK-3*β*, and nuclear *β*-catenin stabilization and increased cyclin D1 transcription were demonstrated in MDA-MB-453 cells [7]. GSK-3*β* acts as a key element in the Wnt/*β*-catenin signaling pathway by dictating cell fate during embryogenesis and tumorigenesis [35]. The Wnt/*β*-catenin signaling pathway has been shown to play an important role in the regulation of cyclin D1, which plays a crucial role in cell-cycle regulation and progression in a variety of tumor cells [20]. The two genes with particular significance for breast cancer are HER-2*/neu *(*erbB2*) and cyclin D1. Both genes have prognostic significance because they are frequently overexpressed and implicated in experimental models of breast cancer [22]. Recent studies clearly described that the interactions between HER-2*/neu* and cyclin D1 appear to have therapeutic relevance because several phytochemical or synthetic drugs reduced cyclin D expression through the inhibition of HER-2/*neu*, and the anti-HER-2*/neu* monoclonal antibody trastuzumab (Herceptin) reduces cyclin D1 protein levels in human breast cancer cells [36, 37]. In addition, our results demonstrated that AC treatment significantly inhibited MDA-M-453 proliferation, which was associated with the suppression of GSK-3*β* and *β*-catenin expression and decreased their transcriptional targets, including cyclin D1 and Cdk4.

Eukaryotic cell-cycle progression is coordinated by the sequential activation of Cdks (cyclin-dependent kinases), the activation of which is dependent upon association with cyclins. Our study proposed that the marked reduction of cyclin D1 levels observed upon the inhibition of Cdk4 followed by HER-2/*neu* supports a critical role for this Cdk4 in HER-2*/neu*-mediated cell-cycle progression. The treatment of HER-2/*neu*-overexpressing breast cancer cells with AC downregulated Cdk4 without altering the Cdk1/2 protein. Based on these results, we believe that AC-induced growth inhibition occurred in the G1-S phase transition. A notably similar result was observed in basal HER-2/*neu*-expressing cell lines, such as MCF-7 and MDA-MB-231 [14, 16]. Cell-cycle progression is also regulated by the relative balance between the cellular concentrations of Cdk inhibitors, including p27^Kip1^ and p21^WAF1^ [16]. In fact, p27^Kip1^ was originally identified in cells arrested by transforming growth factor-*β*. Subsequent studies showed that p27^Kip1^ is a typical Cdk inhibitor and a potential tumor suppressor gene [38]. Previous investigations demonstrated that the downregulation of p27^Kip1^ protein is frequently observed in human cancers, including breast, lung, prostate, gastric, skin, colon, and ovarian cancer, and is usually correlated with poor clinical outcome [15]. Recent studies revealed that HER-2*/neu* induces the downregulation of p27^Kip1^ *via* two independent molecular mechanisms [23, 39]. HER-2*/neu* acted through Akt and GSK-3*β* to reduce p27^Kip1^ protein levels. GSK-3*β* may phosphorylate cyclin D1 and induce the degradation of the p27^Kip1^ protein. The activation of Akt by HER-2*/neu* inhibits GSK-3*β* activity and increases the formation of the cyclin D1/Cdk4 complex, which may sequester p27^Kip1^ in the cytoplasm to enhance its turnover [15]. Also, p21^WAF1^ has been shown to function as an apoptosis-promoting protein, and the mechanisms by which p21^WAF1^ promote apoptosis may be related to its interaction with the DNA repair machinery [40]. Results from the present study showed that the protein expression levels of p27^Kip1^ and p21^WAF1^ are dose-dependently augmented, whereas cyclin D/Cdk4 levels were inhibited by AC treatment. Taken together, we believed that the inhibition of cyclin D/Cdk4 activity may be associated with the augmentation of p27^Kip1^/p21^WAF1^. In addition, we hypothesized that the induction of apoptosis may also be mediated by the activation of p27^Kip1^/p21^WAF1^. However, the role of p21^WAF1^ in apoptosis remains controversial and merits further investigation.

Apoptosis-inducing agents are being investigated as tools for the management of cancer treatment. Apoptosis is characterized by a number of well-defined features, including cellular morphological changes, chromatin condensation, internucleosomal DNA cleavage and the activation of a family of cysteine-aspartic acid proteases (caspases) [41]. In the present study, TUNEL assays demonstrated that treatment of MDA-MB-453 and BT-474 cells with AC markedly induced apoptotic cell death associated with internucleosomal DNA fragmentation. Cells undergoing apoptosis were found to have elevated levels of cytochrome *c *in the cytosol with a corresponding decrease in the mitochondria [14]. Cytosolic cytochrome *c *activates procaspase-9 by binding to Apaf1 in the presence of dATP, leading to the activation of caspase-9 and, subsequently, downstream effector caspases (including caspase-3), triggering apoptosis [42]. In mammalian cells, the Bcl-2 gene family contains a number of antiapoptotic proteins, including Bcl-2 and Bcl-xL, which is thought to be involved in resistance to conventional cancer treatment. However, proapoptotic proteins from the same gene family, including Bax, can critically induce apoptotic cell death. Therefore, apoptosis largely depends on the balance between antiapoptotic and proapoptotic protein levels [43]. We have previously demonstrated that the induction of apoptosis by AC in human breast cancers is associated with Bax protein expression [14, 15]. Similarly, the present study indicates a dose-dependent inhibition of the antiapoptotic protein Bcl-2 and a concomitant increase in the expression of the proapoptotic protein Bax. These data indicate that AC treatment disturbs the Bcl-2/Bax ratio and thereby leads to apoptosis of HER-2*/neu*-overexpressing breast cancer cells. Therefore, we strongly suggest that AC may enhance susceptibility to apoptosis in HER-2*/neu*-overexpressing breast cancer cells.

Anchorage-independent growth is a characteristic of many tumor cells that distinguishes them from their normal counterparts [44]. In stratifying normal epithelium, proliferation is largely confined to the basal layer of cells attached to the basement membrane, which undergo terminal differentiation as they move to the suprabasal layers [45]. This anchorage-dependent growth requires integrin-mediated signaling generated by cellular contact with extracellular matrix ligands [46]. Normal cells, especially epithelial cells, undergo apoptosis if they become detached from their underlying or pericellular matrices, which are a process sometimes, termed anoikis [44]. By contrast, many tumor and transformed cells have escaped this requirement for survival and growth. Moreover, the ability of HER-2/*neu*-overexpressing breast cancer cells to grow in an anchorage-independent manner has been linked to elevation of the PI3K/Akt cell survival pathway [47]. In this study, we found that AC decreased MDA-MB-453 cell proliferation and markedly reduced their capacity to form colonies in soft agar. The loss of anchorage-independent growth of HER-2/*neu*-overexpressing breast cancer cells treated with AC indicates that these cells may have reverted to a less transformed phenotype. This inhibition may also be mediated by the reduction of PI3K/Akt activation.

There is a growing body of evidence that the compounds identified from AC are predominantly polysaccharides, triterpenoids, steroids, benzenoids, and maleic/succinic acid derivatives [11–13]. The reported yields of polysaccharides, crude triterpenoids, and total polyphenols in the fermented AC broth were 23.2 mg/g, 47 mg/g, and 67 mg/g, respectively, whereas no polysaccharides, crude triterpenoids, or polyphenols were detected in the dry matter of the culture medium [18]. Yeh et al. demonstrated that five lanostanes (dehydroeburicoic acid, 15*α*-acetyl dehydrosulfurenic acid, 24-triene-21-oic acid, dehydrosulfurenic acid, and sulfurenic acid) and three ergostane-type triterpenes (zhankuic acid, zhankuic acid-A, and zhankuic acid-C) isolated from fruiting bodies of AC exhibit *in vitro* antiproliferative effects against various cancer cell lines, including MDA-MB-231 [48]. Zhankuic acid and sulfurenic acid had significant cytotoxic effects in the human breast cancer cells MDA-MB-231 and MCF-7, with IC_50_ values of 25.1 and 89.2 and 57.8 and 357.0 *μ*M, respectively, being observed [48]. Antroquinonol, an ubiquinone derivative that was isolated from the solid-state fermented mycelium of AC, exhibits a cytotoxic effect against MDA-MB-231 and MCF-7 human breast cancer cells with an IC_50_ of 2.64 and 2.1 *μ*M, respectively [49]. Furthermore, chloroform extracts of the fruiting bodies of AC significantly inhibited the growth of human breast cancer (MCF-7) cells with an IC_50_ of 65 *μ*M [50]. A notably similar result was obtained with another pure compound, antrocin, which was isolated from the fruiting bodies of AC and exhibited the highest antiproliferative effect against MDA-MB-231 and MCF-7 cells [17]. Notably, nontumorigenic breast epithelial MCF-10A cells were not affected by antrocin treatment. Previous studies have shown that naturally derived phytocompounds downregulate HER-2/*neu *expression at both the transcriptional and translational levels, eventually suppressing tumor growth and dissemination [7, 8]. In this study, we demonstrated that the fermented culture broth of AC exhibited significant growth inhibition that was followed by the inhibition of HER-2/*neu* and tyrosine phosphorylation in HER-2/*neu*-overexpression breast cancer cells. It is reasonable to suggest, therefore, that AC metabolizes the culture medium and releases active components during fermentation by submerged culture. Further bioassay-directed fractionations leading to the identification and purification of the compounds responsible for the anti-breast-cancer effect of AC are warranted.

In this study, we proposed that AC induced cellular effects resulting from ROS generation and loss of HER-2/*neu *expression with subsequent inactivation of PI3K and Akt in cells that are dependent on this pathway for cell proliferation and inhibition of apoptosis. Our results also highlight the importance of HER-2/*neu* or PI3K/Akt components, including GSK-3*β*, *β*-catenin, cyclin D1, Cdk4, p21^WAF1^, and p27^KIP1^, which may serve as future targets for the development of therapeutic strategies against HER-2/*neu*-overexpressing breast cancer. To the best of our knowledge, this is the first study to focus on the effect of *Antrodia camphorata* on HER-2/*neu* signaling components in breast cancer. The inhibition of cell proliferation and induction of apoptosis in HER-2/*neu-*overexpressing breast cancer cells upon *Antrodia camphorata* administration provides a new strategy for breast cancer treatment. However, *in vivo* studies are needed to confirm the pharmacological efficacy and safety of *Antrodia camphorata*.

## References

[1] Ferlay J, Shin HR, Bray F, Forman D, Mathers C, Parkin DM (2010). Estimates of worldwide burden of cancer in 2008: GLOBOCAN 2008. *International Journal of Cancer*.

[2] Chang SB, Miron P, Miron A, Iglehart JD (2007). Rapamycin inhibits proliferation of estrogen-receptor-positive breast cancer cells. *Journal of Surgical Research*.

[3] Ao A, Morrison BJ, Wang H, López JA, Reynolds BA, Lu J (2011). Response of estrogen receptor-positive breast cancer tumorspheres to antiestrogen treatments. *PLoS One*.

[4] Shah S, Chen B (2011). Testing for HER2 in breast cancer: a continuing evolution. *Pathology Research International*.

[5] Cho HS, Mason K, Ramyar KX (2003). Structure of the extracellular region of HER2 alone and in complex with the Herceptin Fab. *Nature*.

[6] Nunes RA, Harris LN (2002). The HER2 extracellular domain as a prognostic and predictive factor in breast cancer. *Clinical Breast Cancer*.

[7] Way TD, Kao MC, Lin JK (2004). Apigenin induces apoptosis through proteasomal degradation of HER2/*neu* in HER2/*neu*-overexpressing breast cancer cells via the phosphatidylinositol 3-kinase/Akt-dependent pathway. *Journal of Biological Chemistry*.

[8] Jeong JH, Jee YA, Yong TK, Li LY, Lee YJ (2008). Quercetin-induced ubiquitination and down-regulation of Her-2/*neu*. *Journal of Cellular Biochemistry*.

[9] Zang M, Su CH (1990). Ganoderma comphoratum, a new taxon in genus Ganoderma from Taiwan, China. *Acta Botanica Yunnanica*.

[10] Tsai ZT, Liaw SL (1985). *The Use and the Effect of Ganoderma*.

[11] Ao ZH, Xu ZH, Lu ZM, Xu HY, Zhang XM, Dou WF (2009). Niuchangchih (*Antrodia camphorata*) and its potential in treating liver diseases. *Journal of Ethnopharmacology*.

[12] Geethangili M, Tzeng YM (2011). Review of pharmacological effects of *Antrodia camphorata* and its bioactive compounds. *Evidence-Based Complementary and Alternative Medicine*.

[13] Yang HL, Kumar KJS, Hseu YC Multiple Molecular Targets of Antrodia camphorata: A Suitable Candidate for Breast Cancer Chemoprevention. *Breast Cancer Cells-2*.

[14] Yang HL, Chen CS, Chang WH (2006). Growth inhibition and induction of apoptosis in MCF-7 breast cancer cells by Antrodia camphorata. *Cancer Letters*.

[15] Hseu YC, Chen SC, Tsai PC (2007). Inhibition of cyclooxygenase-2 and induction of apoptosis in estrogen-nonresponsive breast cancer cells by *Antrodia camphorata*. *Food and Chemical Toxicology*.

[16] Hseu YC, Chen SC, Chen HC, Liao JW, Yang HL (2008). Antrodia camphorata inhibits proliferation of human breast cancer cells in vitro and in vivo. *Food and Chemical Toxicology*.

[17] Rao YK, Wu ATH, Geethangili M (2011). Identification of antrocin from antrodia camphorata as a selective and novel class of small molecule inhibitor of Akt/mTOR signaling in metastatic breast cancer MDA-MB-231 cells. *Chemical Research in Toxicology*.

[18] Hseu YC, Huang HC, Hsiang CY (2010). Antrodia camphorata suppresses lipopolysaccharide-induced nuclear factor-*κ*B activation in transgenic mice evaluated by bioluminescence imaging. *Food and Chemical Toxicology*.

[19] Koleske AJ, Baltimore D, Lisanti MP (1995). Reduction of caveolin and caveolae in oncogenically transformed cells. *Proceedings of the National Academy of Sciences of the United States of America*.

[20] Takahashi-Yanaga F, Sasaguri T (2008). GSK-3*β* regulates cyclin D1 expression: a new target for chemotherapy. *Cellular Signalling*.

[21] Mortenson MM, Galante JM, Schlieman MG, Bold RJ (2004). AKT: a novel target in pancreatic cancer therapy. *Cancer Therapy*.

[22] Way TD, Kao MC, Lin JK (2005). Degradation of HER2/*neu* by apigenin induces apoptosis through cytochrome *c* release and caspase-3 activation in HER2/*neu*-overexpressing breast cancer cells. *FEBS Letters*.

[23] Hseu YC, Chen SC, Yech YJ, Wang L, Yang HL (2008). Antioxidant activity of Antrodia camphorata on free radical-induced endothelial cell damage. *Journal of Ethnopharmacology*.

[24] Yang HL, Hseu YC, Chen JY (2006). Antrodia camphorata in submerged culture protects low density lipoproteins against oxidative modification. *American Journal of Chinese Medicine*.

[25] Hahn T, Bradley-Dunlop DJ, Hurley LH (2011). The vitamin E analog, alpha-tocopheryloxyacetic acid enhances the anti-tumor activity of trastuzumab against HER2/neu-expressing breast cancer anti-tumor activity of trastuzumab against HER2/neu-expressing breast cancer. *BMC Cancer*.

[26] Liu F-S, Yang P-Y, Hu D-N, Huang Y-W, Chen M-J (2011). Antrodia camphorata induces apoptosis and enhances the cytotoxic effect of paclitaxel in human ovarian cancer cells. *International Journal of Gynecological Cancer*.

[27] Ozben T (2007). Oxidative stress and apoptosis: impact on cancer therapy. *Journal of Pharmaceutical Sciences*.

[28] Chen FH, Zhang LB, Qiang L (2010). Reactive oxygen species-mitochondria pathway involved in LYG-202-induced apoptosis in human hepatocellular carcinoma HepG2 cells. *Cancer Letters*.

[29] Frank GD, Mifune M, Inagami T (2003). Distinct mechanisms of receptor and nonreceptor tyrosine kinase activation by reactive oxygen species in vascular smooth muscle cells: role of metalloprotease and protein kinase C-*δ*. *Molecular and Cellular Biology*.

[30] Franke TF, Hornik CP, Segev L, Shostak GA, Sugimoto C (2003). PI3K/Akt and apoptosis: size matters. *Oncogene*.

[31] Zhang X, Jin B, Huang C (2007). The PI3K/Akt pathway and its downstream transcriptional factors as targets for chemoprevention. *Current Cancer Drug Targets*.

[32] Datta SR, Brunet A, Greenberg ME (1999). Cellular survival: a play in three akts. *Genes and Development*.

[33] Zheng L, Ren JQ, Li H, Kong ZL, Zhu HG (2004). Downregulation of wild-type p53 protein by HER-2/*neu* mediated PI3K pathway activation in human breast cancer cells: Its effect on cell proliferation and implication for therapy. *Cell Research*.

[34] Basso AD, Solit DB, Munster PN, Rosen N (2002). Ansamycin antibiotics inhibit Akt activation and cyclin D expression in breast cancer cells that overexpress HER2. *Oncogene*.

[35] Fishman P, Madi L, Bar-Yehuda S, Barer F, Del Valle L, Khalili K (2002). Evidence for involvement of Wnt signaling pathway in IB-MECA mediated suppression of melanoma cells. *Oncogene*.

[36] Smith-Schneider S, Roberts LA, Shetty K, Shetty K, Paliyath G, Pometto AL, Levin RE (2005). Phytochemicals and Breast Cancer Chemoprevention. *Food Biotechnology*.

[37] Xu J, Chen Y, Olopade OI (2010). MYC and Breast Cancer. *Genes & Cancer*.

[38] Denicourt C, Dowdy SF (2004). Cip/Kip proteins: more than just CDKs inhibitors. *Genes and Development*.

[39] Yeh CT, Yao CJ, Yan JL (2011). Apoptotic cell death and inhibition of Wnt/*β*-catenin signaling pathway in human colon cancer cells by an active fraction (hs7) from *Taiwanofungus camphoratus*. *Evidence-Based Complementary and Alternative Medicine*.

[40] Gartel AL, Tyner AL (2002). The role of the cyclin-dependent kinase inhibitor p21 in apoptosis. *Molecular cancer therapeutics*.

[41] Casaccia-Bonnefil P (2000). *GLIA*.

[42] Franklin EE, Robertson JD (2007). Requirement of Apaf-1 for mitochondrial events and the cleavage or activation of all procaspases during genotoxic stress-induced apoptosis. *Biochemical Journal*.

[43] Coultas L, Strasser A (2003). The role of the Bcl-2 protein family in cancer. *Seminars in Cancer Biology*.

[44] Ghatak S, Misra S, Toole BP (2002). Hyaluronan oligosaccharides inhibit anchorage-independent growth of tumor cells by suppressing the phosphoinositide 3-kinase/Akt cell survival pathway. *Journal of Biological Chemistry*.

[45] Kantak SS, Kramer RH (1998). E-cadherin regulates anchorage-independent growth and survival in oral squamous cell carcinoma cells. *Journal of Biological Chemistry*.

[46] Millard M, Odde S, Neamati N (2011). Integrin targeted therapeutics. *Theranostics*.

[47] Menendez JA, Mehmi I, Verma VA, Teng PK, Lupu R (2004). Pharmacological inhibition of fatty acid synthase (FAS): a novel therapeutic approach for breast cancer chemoprevention through its ability to suppress Her-2/*neu* (erbB-2) oncogene-induced malignant transformation. *Molecular Carcinogenesis*.

[48] Yeh CT, Rao YK, Yao CJ (2009). Cytotoxic triterpenes from *Antrodia camphorata* and their mode of action in HT-29 human colon cancer cells. *Cancer Letters*.

[49] Lee TH, Lee CK, Tsou WL, Liu SY, Kuo MT, Wen WC (2007). A new cytotoxic agent from solid-state fermented mycelium of *Antrodia camphorata*. *Planta Medica*.

[50] Rao YK, Fang SH, Tzeng YM (2007). Evaluation of the anti-inflammatory and anti-proliferation tumoral cells activities of *Antrodia camphorata, Cordyceps sinensis*, and *Cinnamomum osmophloeum* bark extracts. *Journal of Ethnopharmacology*.

